# Evaluating the consequences of health policy decisions

**DOI:** 10.1186/s13584-020-00380-7

**Published:** 2020-04-30

**Authors:** Ulrike Nimptsch

**Affiliations:** Fachgebiet Management im Gesundheitswesen, Technische Universität Berlin, Straße des 17. Juni 135, 10623 Berlin, Germany

## Abstract

Uncertainty about intended and possible unintended side effects makes it important to evaluate changes following health policy decisions. A recent IJHPR article by Greenberg et al. evaluated changes in emergency department care following a directive of the Israeli Ministry of Health to limit occupancy in internal medicine wards. Over a six-year observation period, they found that one-month mortality and one-week readmissions after ED visits remained unchanged, while increases in average ED visit length, as well as increased delay time from ED admission to ward were observed. These findings help to assess the impact of the occupancy limit directive and may support future health policy decisions.

However, the study by Greenberg et al. was limited by the unavailability of diagnostic data, and this illustrates a significant issue that transcends this particular study. In many countries, policy-relevant administrative data are not sufficiently available on a timely basis. Data availability is the prerequisite for monitoring developments in patterns of care following health policy changes. Besides conducting retrospective studies, timely availability of data makes it possible to establish monitoring systems which may help decision makers assess the impact of policy changes, identify undesired developments early, and recognize changes in need or demand of health services within the population.

## Main text

Health policy decisions often aim to strike a balance between the allocation of limited resources on the one hand, and high quality provision of care on the other hand. Besides the intended effects of health policy decisions, unintended side effects might occur, which are often not fully predictable. This uncertainty makes it important to evaluate changes following health policy decisions by focusing on both intended and possible unintended consequences.

Greenberg et al. [1] investigated changes in emergency department care following a directive of the Israeli Ministry of Health to limit occupancy in internal medicine wards. They analyzed administrative data from the Tel Aviv Medical Center Internal Emergency Medicine Department (ED) and studied readmission rates and mortality as primary outcomes. They found that until 2016 the number of ED visits increased, whereas there was a decline in the number of ED cases that were admitted as inpatients. Overall, bed occupancy in the internal medicine wards decreased, as intended by the directive to limit occupancy. One-month mortality and one-week readmissions after ED visits remained unchanged during the observation period, suggesting that the occupancy limit directive did not affect quality of care, with regard to those indicators. However, the authors further analyzed patterns of ED care and observed increases in average ED visit length, as well as increased delay time from ED admission to ward, which they assessed as being probably related to the observed higher ED crowding. From those findings and in view of international research the authors propose interventions such as proper adjustments in ED size and personnel.

The work of Greenberg et al. is a well conducted study and provides figures and findings that help decision makers to assess the impact of the occupancy limit directive and, thus, may support future health policy decisions. However, expanding the analysis to all Israeli hospitals might provide additional findings, since strategies of different hospitals following the policy change might differ and subsequent changes and consequences may vary between hospitals or regions.

The authors state that from their retrospective study no causal relation could be made between the policy change and the findings of their study. This is a common limitation of health policy evaluation studies when policy changes are implemented in the entire health system at the same time and, thus, no control group is available. Nevertheless, monitoring and evaluation are essential wherever there is uncertainty about the effects of health system interventions [2, 3]. Even if there is no proof of causality, data-based investigation of trends is the only way to detect undesirable developments and, thus, to enable policy makers to respond appropriately.

Another limitation of the study by Greenberg et al. is that no diagnostic data were available. It might have been instructive to see which diagnoses were associated with hospital admissions and which diagnoses were not. Beyond that, identification of conditions representing unnecessary visits to the emergency department may hold lessons for ambulatory care and thus, may support health policy interventions that aim to prevent emergency department overcrowding.

Data availability is the prerequisite for monitoring developments in patterns of care following health policy changes. Nowadays most health systems routinely collect administrative data which are a proper source for studying real world practice of care. Besides conducting retrospective studies, availability of reliable administrative data in a timely manner allows to establish monitoring systems which may help decision makers assess the impact of policy changes, identify undesired developments early, and recognize changes in need or demand of health services within the population. Although the benefit of available data could be considered as undisputable, in many industrialized countries there is a lack of data itself or availability of data is timely delayed. In Germany, for example, there is no standardized national data collection on emergency department services available, to date [4]. National complete inpatient data from hospitals, however, is available, but the data are provided for research only with a delay of at least 2 years [5].

Most recently, health systems in many countries face the challenge of coping with the COVID-19 pandemic outbreak. This sheds a new light on the need for timely data availability and monitoring systems, which are now an imperative requirement for tracking the capacity and occupancy of hospital beds and for responding appropriately to rapidly growing needs for inpatient capacity. In the future, retrospective studies of real-world data will help to understand how health systems responded to this unprecedented burden of care and may help to create prospective policy plans for possible future occurrences of unpredicted large-scale needs in acute health care.

## Data Availability

Not applicable.
